# Housing Stability and Hepatitis C Infection for Young Adults Who Inject Drugs: Examining the Relationship of Consistent and Intermittent Housing Status on HCV Infection Risk

**DOI:** 10.1007/s11524-020-00445-7

**Published:** 2020-09-08

**Authors:** Meghan D. Morris, Irene H. Yen, Steve Shiboski, Jennifer L. Evans, Kimberly Page

**Affiliations:** 1 Department of Epidemiology and Biostatistics, School of Medicine, University of California, San Francisco, 550 16th Street, Box 1224, San Francisco, CA 94153-1224 USA; 2 Department of Public Health, School of Social Sciences, Humanities & Arts, University of California, Merced, Merced, CA USA; 3 Department of Internal Medicine, School of Medicine, University of New Mexico Health Sciences Center, MSC10 5550, 1 University of New Mexico, Albuquerque, NM 87131 USA

**Keywords:** Hepatitis C virus, Housing status, People who inject drugs

## Abstract

Housing status affects drug using behaviors, but less is known about the relationship between housing patterns and hepatitis C virus (HCV) infection. HCV-negative young people who inject drugs (PWID) were enrolled into a prospective cohort (2003–2019) with quarterly study visits. We used Cox regression to estimate the independent association of recent housing status (housed vs. unhoused, housing stability, and housing trajectory) on HCV incidence. Among 712 participants, 245 incident HCV infections occurred over 963.8 person-years (py) (cumulative incidence 24.4/100 py). An inverse relationship between time housed and HCV incidence was observed (always unhoused 45.0/100 py, 95% confidence interval (CI) 37.1, 54.5; variably housed 18.0/100 py, 95% CI 15.0, 21.3; and always housed 7.0/100 py, 95% CI 3.0, 17.3). In Cox regression models controlling for confounders, those unhoused versus housed at baseline had a 1.9-fold increased infection risk (95% CI 1.4, 2.6). Those always unhoused versus always housed had a 1.5 times greater risk of HCV (95% CI 1.0, 2.3), and those spending a portion of time in stable housing a lower risk (adjusted relative hazard 0.05, 95% CI 0.3, 0.9) with a similar trend for those being housed for less time. Young adult PWID experiencing both recent and chronic states of being unhoused are at elevated risk for HCV infection. Importantly for this group of PWID, our findings indicate that some frequency of residential housing significantly reduces HCV infection risk.

**Electronic supplementary material:**

The online version of this article (10.1007/s11524-020-00445-7) contains supplementary material, which is available to authorized users.

## Introduction

Housing offers more than a physical space to live. Housing offers people security, privacy, and the possibility to create relationships [1]. In contrast, being unhoused can introduce stress and increase social and physical vulnerability [2–4]. Housing therefore is considered a key social determinant of health [5].

Studies have shown that unhoused populations experience worse health outcomes. A study of people entering a New York City homeless shelter found residents had higher rates of medical illness, psychopathology, and substance use disorders when compared with a general population of similar age [6]. Another study in Los Angeles County reported lower cancer screening rates among adults experiencing homelessness compared with the general California population, despite having higher rates of cancer risk factors [7]. In reproductive health, homelessness and frequent relocation have been associated with higher risk for low birth weight [8, 9]. For people living with HIV, homelessness has been associated with delayed HIV care access, poorer HIV medical care, and lower adherence to antiretroviral therapy [3]. Receiving housing assistance is associated with having health insurance, translating to increased medical treatment access, reduced medical service access for acute conditions, and improved depressive symptoms [10, 11].

Housing may also impact substance use behavior. A study of chronically homeless individuals with alcohol problems found that a “Housing First” approach—here individuals were provided permanent supportive housing and opportunities (but no requirement) for substance use support—reported alcohol dependence symptoms decreased by 4% every 3 months [12]. Another Seattle “Housing First” study found that services costs (including hospital-based medical services, substance use treatment, days incarcerated, shelter use, and Medicaid-funded services) more than halved after 12 months of housing assistance resulting in an average overall decrease in costs of $2450 per person per month [13].

A health outcome for which housing may be particularly relevant is hepatitis C virs (HCV) infection. HCV prevalence among adults experiencing homelessness in the USA is an estimated 14.7%, compared with 1.5% among the non-homeless US population [14]. HCV prevalence and homelessness have also been linked to injection drug use. HCV infection rates are disproportionately high among US adults who inject drugs (PWID), with San Francisco–based studies estimating that 42% of PWID have chronic HCV infection [15]. People experiencing homelessness are disproportionately more likely to inject drugs, with more than a third of all adults who stay in shelters having chronic substance use issues in 2010 [16].

Studies on acquisition and transmission of HCV infection among PWID frequently include housing status as a baseline measure of demographic characteristics or as an adjustment covariate in regression models [17–19], limiting the interpretation of the association of housing on HCV infection risk. To our knowledge, only one study of PWID in Vancouver, Canada, examined the relationship between housing as a primary exposure and HCV seroconversion [20]. Results demonstrated that people experiencing recent housing instability had a nearly 50% higher hazard for incident HCV than those who were stably housed, controlling for injection drug use. This study provided valuable evidence to establish a connection between recent housing and HCV seroconversion, but focused on one moment within one’s larger housing trajectory. Limiting analyses to recent housing type may miss the association between chronic homelessness or changes in housing trajectories and HCV infection. Homelessness and frequent transitions are more common for highly marginalized populations like PWID. Furthermore, looking only at one housing time point may not capture the additive influence of being homeless [21, 22]. In sum, a binary measure of recent homelessness at a single time point may not capture the dimensionality and fluidity of housing experiences that impact the individuals at highest risk for HCV.

This study aims to explore the relationship between housing and incident HCV infection among PWID living in San Francisco, CA, a city experiencing a housing affordability crisis exacerbating homelessness rates [23], and the presence of a burgeoning HCV epidemic disproportionately affects PWID [15]. We constructed four measures of housing: housing status, housing stability, housing trajectory, and percent-time housed (proportion of participant response data indicating being recently unhoused). We hypothesize that both recent housing (as reflected by housing status and housing stability) and chronic housing (as reflected by housing trajectory and percent-time housed) increased risk for HCV infection, with chronic housing having a stronger association with HCV infection risk. Our study used longitudinal data from a prospective cohort of young adult PWID in San Francisco of HCV acquisition and transmission.

## Methods

### Data Source

This study analyzed longitudinal data from the “UFO Study,” a prospective observational cohort study of hepatitis C virus (HCV) transmission among young adult (< 30 years old) people who inject drugs (PWID) in San Francisco [24, 25], between January 2000 and January 2019. This study was reviewed by the Institutional Review Board at the University of California, San Francisco (No. 10-00063).

### Study Subjects

UFO Study participants were PWID 18–30 years of age without chronic HCV infection (anti-HCV and HCV RNA negative at enrollment) who attended at least one follow-up study visit. Participants completed written consent, a baseline study visit, and quarterly follow-up study visits. Baseline and quarterly follow-up visits included structured interviews and serological tests for both anti-HCV and HCV RNA (described in detail below). Survey questions included items about demographic characteristics, injection drug use–related risk behaviors, access to HCV prevention services, and time spent in jail/prison. Participants were remunerated for all study visits including screening (10 USD) and follow-up (20–25 USD).

### Outcome and Relevant Variables

The primary outcome of interest was incident HCV infection defined as either (1) positive anti-HCV and HCV RNA positive or (2) positive HCV RNA test following a previously documented negative anti-HCV and HCV RNA test. Anti-HCV testing was performed using EIA-3 (Ortho Clinical Diagnostics, Raritan, NJ) and after 2012, using the OraQuick rapid test (OraSure Technologies, Inc., Bethlehem, PA). Qualitative HCV RNA testing was performed on all participants using the Gen-Probe (San Diego, CA)/Chiron (Emeryville, CA) HCV TMA assay. We estimated the HCV RNA infection date as the midpoint of the interval between the last HCV RNA negative test and the first HCV RNA positive test. Total time to infection was the number of days from the first HCV RNA negative test to the estimated infection date. If the first visit was anti-HCV negative and HCV RNA positive, the time to infection was estimated to be 50.8 days [26].

The primary exposure was housing status ascertained at each study visit through a single–response survey item asking “what was the main type of place you lived in the *last 3 months*?” Response answers included: own apartment, parent/relative home, friend’s home, halfway/foster/group home, hotel/boarding house, shelter, squat, park, street/freeway/doorway, vehicle, residential detox, jail, prison, and other.

We constructed four aggregate measures of housing status to assess both the immediate and cumulative influence of homelessness on risk for incident HCV infection. First, *recent housing status* was assessed using a time-varying dichotomous measure reflecting whether an individual was recently (past 3 months) housed versus unhoused. Responses representing being recently housed included: staying in own apartment, parent/relative home, friend’s home, or halfway/foster/group home; being unhoused included: shelter, squat, park, street/freeway/doorway, vehicle, residential detox, jail, or prison. Second, *recent housing stability* was assessed using an ordinal measure that reflected (i) permanent housing (e.g., own apartment/house, parent/relative house, friend’s house, and foster/group home), (ii) temporary housing (e.g., shelter, squat, vehicle, residential detox, jail or prison), and (iii) no housing (e.g., park, street/freeway/doorway, other). Third, to assess the cumulative influence of housing status on HCV incident infection, we constructed two time-varying ordinal summary measures using all available non-missing visit data for participants between study baseline and each study visit up until the time of censoring/infection: (1) *housing trajectory* and (2) *percent-time unhoused*. Housing trajectory was defined as being: always housed, always unhoused, or variably housed, with the latter value representing participants who reported being both housed and unhoused over the observed period. Percent-time housed was calculated as the proportion of participant response data indicating being recently unhoused over their total number of responses for that period.

### Adjustment Variables

Sociodemographic variables included as potentially confounding factors in the analyses included age at enrollment, self-reported gender (male, female, transgender), race/ethnicity (White vs. non-White), educational level (less than high school vs. high school or greater), born outside California (yes/no), primary source of income in past month, past 3-month incarceration, and past 3-month medical care access. Lifetime and past 3-month illicit drug use included questions about injecting frequency, main drug injected in past month, number of injecting partners, and injecting behaviors (receptive and distributive sharing of needles/syringes, equipment, and drug residue (rinse)). Frequency of recent alcohol use was also assessed. Information was collected about lifetime and recent use of medically assisted treatment (MAT) for drug dependence and syringe service programs (SSPs). Lastly, we assessed the role of social support indicated by a measure of recently living with one’s injecting partner (yes/no).

### Statistical Analyses

To assess potential bias due to differential loss to follow-up, we used Pearson *x*^2^ or Kruskal-Wallis test to compare distributions of selected demographic and injecting risk behavior variables between participants with follow-up and those with only baseline visits. To summarize variability in housing status over a participant’s observation time, we constructed a housing heatmap showing one’s transition from a housed status to an unhoused status (or vice versa). To capture temporal changes in housing across the cohort waves, we constructed a graph depicting the proportion of study participants across thirteen housing types at each cohort wave (2000–2002, 2003–2008, 2009–2013, 2015–2019).

We used descriptive statistics to characterize the study population overall and by HCV incident infection status. The Kaplan-Meier method was used to describe the cumulative incidence of HCV infection separately in groups differentiated by values for (a) recent housing status (housed vs. unhoused), (b) recent housing stability (permanent, temporary, none), (c) housing trajectory (always housed, always unhoused, variable housing), and (d) percent-time housed (≤ 25% time unhoused, 26–50% time unhoused, 51–75% time unhoused, and ≥ 76% time unhoused). Differences between groups were evaluated using the log-rank test.

Hazard ratios (HR), adjusted hazard ratios (aHR), and 95% confidence intervals (CI) were estimated for the housing groups using bivariate and multivariate Cox proportional hazard regression models used to estimate the risk of an event occurrence within a specified time interval, conditional on survival to the beginning of that time interval. For example, people who become infected with HCV or were lost to follow-up in the first 3 months after enrollment are not included in calculation of the hazard rate for the second or subsequent observation periods. One at a time, we included each variable found to have a significant relationship in bivariate models along with the housing measure as the only other covariate and examined the change in the adjusted hazard ratio (aHR) and 95% CI. Any variable that changed the adjusted hazard ratio and was statistically significant was considered a confounder. Potential explaining variables tested in the models included gender, age, education level, number of years injecting drugs, recent jail/prison, injecting frequency, recent cooker/container sharing, recent needle/syringe sharing, recent backloading/piggybacking, recent rinse use, number of injecting partners, and recent income source. The recall period for recent behaviors was 3 months and measures were modeled as time-varying covariates in regression models. Due to collinearity, we collapsed recent cooker/container, needle/syringe sharing, and backloading/piggybacking together into “recent needle/syringe or ancillary equipment sharing.” The variables included in the multivariable model included age at enrollment, gender, recent injecting frequency, recent needle/ancillary equipment sharing, and recent number of injecting partners as these are conceptualized as confounders in the relationship between housing and HCV risk. Recent drug using behaviors were collected quarterly and included in the model as time-varying covariates.

We restructured the data to follow the intended 3-month follow-up interval and carried forward the previous value for any participants with missing housing and adjustment variables. All analyses were performed in Stata 15.

## Results

Between January 2000 and June 2019, 904 eligible young adult PWID were enrolled into the UFO Study. Among these participants, 712 (79%) had at least one follow-up visit, representing a total of 982.67 person-years (py) of follow-up. The median duration of follow-up was 9.12 months (interquartile range (IQR) 2.96–22.56 months). The majority of participants were male (67%), White (71%), and born outside of the study state of California (62%), and the median age was 24 years at baseline (IQR 21–26). Most participants self-reported being HIV-negative (96%), recent alcohol use (75%), and injecting drugs multiple times per week (median number of days per month 23, IQR 10–30), with more than half (63%) reporting heroin/opioids as the main drug injected and one-third (33%) injecting amphetamines/cocaine/crack most often. Compared with the analytic sample, those lost to follow-up and not included in analysis differed significantly in age, education, recent alcohol and drug use frequency, and recent incarceration, with the analytical sample being older in age and less risky than those not returning for follow-up study visits (Supplemental Table 1).

The housing context for young adult PWID in San Francisco: With respect to housing status, at baseline, 70% (*n* = 501) reported being unhoused and 30% (*n* = 211) reported being housed. With respect to housing stability, at baseline, 30% (*n* = 219) reported living in permanent housing, 27% (*n* = 192) reported living in a temporary form of housing, and 43% (*n* = 312) reported being unhoused. Additional baseline characteristics are shown in Table 1, among all participants and by incident HCV status.

**Table 1:** Demographic characteristics of HCV-negative young adult people who inject drugs and unadjusted hazard ratios at baseline (*n* = 712)

Characteristic	Overall		No HCV infection	HCV infection	*p* value^†^
	*N*	%	%	%	
Baseline housing type					< 0.001
Own home	91	13.06	15.46	7.93	
Parent/relative’s home	25	3.51	3.92	2.64	
Friend’s home	88	13.06	14.43	10.13	
Foster/group home	7	0.84	1.24	0	
Hotel/boarding house	80	11.66	12.99	8.81	
Shelter	23	3.23	3.92	1.76	
Squat	68	9.69	9.28	10.57	
Park	61	8.71	6.8	12.78	
Street/freeway/doorway	207	27.81	22.68	38.77	
Vehicle	20	2.81	3.71	0.88	
Residential detox	4	0.56	0.62	0.44	
Jail/prison	13	1.82	2.04	1.22	
Other	25	3.23	2.89	3.96	
Baseline housing status					< 0.001
Housed	211	30.00	35.05	20.7	
Unhoused	501	70.00	64.95	79.3	
Baseline housing type					< 0.001
Housed	211	30.00	34.9	19.59	
Temporary housing	188	26.00	28.69	22.04	
Unhoused	313	44.00	36.4	58.37	
Gender					0.079
Male	485	67.30	68.93	63	
Female	227	31.50	29.42	36.56	
Transgender	9	1.20	1.65	0.44	
Age at baseline					0.003
22 years old or younger	261	36.61	67.08	55.51	
Over 22 years old	452	63.39	32.92	44.49	
Ethnicity					0.183
White	511	70.9	69.14	74.01	
Non-White	210	29.1	30.86	25.99	
Education level					0.020
High school or higher	459	64.1	67.08	58.15	
Less than high school	257	35.9	32.92	41.85	
Born in California					0.323
Yes	268	38.2	39.79	35.87	
No	433	61.8	60.21	64.13	
Recent medical care (3 months)*					0.916
Yes	416	57.8	57.73	58.15	
No	304	42.2	42.27	41.85	
HIV status					0.347
Positive	22	3.1	3.53	2.21	
Negative	694	96.3	96.47	97.79	
Ever been tested for HCV (*n* = 643)**					0.429
Yes	418	65	64.41	66.15	
No	207	32.2	32.21	32.24	
Unknown	18	2.8	3.38	1.54	
Opiod pill use ever (methadone, buprenorphine, suboxone, fentanyl, codeine, vicodine, darocet, percocet, percodan, dilaudid, morphine pills)					0.683
Yes	637	89.2	88.8	89.82	
No	77	10.8	11.2	10.18	
Opioid pill use in past 3 months					0.959
Yes	453	62.8	62.76	62.56	
No	268	37.2	37.24	37.44	
Alcohol use in past 3 months					0.584
Yes	544	75.5	76.34	74.45	
No	177	24.5	23.66	25.55	
Age first injected drugs					
Median (IQR)	18.8	16.0–21.4	19 (16.75, 22.00)	18 (16, 21)	
Age first injected drugs					0.068
Younger than 18 years old	394	54.7	57.00	49.79	
Older than 18 years old	327	45.4	43.00	50.21	
Years injecting drugs					
Median (IQR)	3.7	1.7–7.0	3.75 (1.60, 6.910	3.71 (1.71, 7.31)	
Recent jail/prison in past 3 months					0.118
Yes	193	26.7	24.95	30.53	
No	522	73.3	75.05	69.47	
Main drug injected past month					0.021
Heroin/heroin mixed with other drugs	447	62.9	59.45	69.60	
Meth/cocaine/crack	232	32.6	35.08	27.75	
Other	32	4.5	5.46	2.64	
Number of days injected in past month					
Median (IQR)	23	10–30	20 (7, 30)	29 (15, 30)	
Injected everyday in the past month					< 0.001
Yes	257	36.04	30.45	48.02	
No	456	63.96	69.55	51.98	
Ever shared cooker/container in past 3 months					0.004
Yes	424	58.9	55.26	66.52	
No	296	41.1	44.74	33.48	
Ever borrowed used needle/syringe in past 3 months					0.004
Yes	247	34.5	30.91	41.85	
No	470	65.5	69.09	58.15	
Ever backloaded/piggybacked in past 3 months					< 0.001
Yes	445	62.0	57.32	72.44	
No	273	38.0	42.68	27.56	
Ever done someone’s rinse in past 3 months					< 0.001
Yes	275	38.3	32.44	50.66	
No	444	61.7	67.56	49.34	
Ever lent used rig in past 3 months (*n* = 411)***					< 0.001
Yes	159	38.7	32.99	54.46	
No	252	61.3	67.01	45.54	
Number of injecting partners in past 3 months					
Median (IQR)	5	2–11	4 (1, 10)	10 (4, 19)	
Live with injecting partner month (*n* = 401)***					0.114
Yes	66	16.5	18.73	12.40	
No	335	83.5	81.27	87.60	
Personally got new rigs from NSP in past 3 months					0.004
Yes	563	78.3	75.00	84.58	
No	156	21.7	25.00	15.42	
Recent overdose in past 3 months					0.081
Yes	97	13.5	6.20	3.08	
No	620	86.5	93.80	96.92	
Recent OAT in past 3 months					0.097
Yes	39	5.4	12.22	16.81	
No	680	94.6	87.78	83.19	
Recent sex trade in past 3 months					0.564
Yes	99	13.7	13.37	14.98	
No	622	83.3	86.63	85.02	
Recent income source from legal source					0.001
Yes	198	27.7	31.69	19.38	
No	515	72.3	68.31	80.62	
Recent travel					0.148
Yes	329	46.3	44.42	50.22	
No	382	53.7	55.58	49.78	

*Only collected 2014–2018. **Not collected 2003–2005. ***Not collected 2000–2005

^†^The *p* value reflects a comparison of characteristics by HCV incident infection groups

**Fig. 1:**
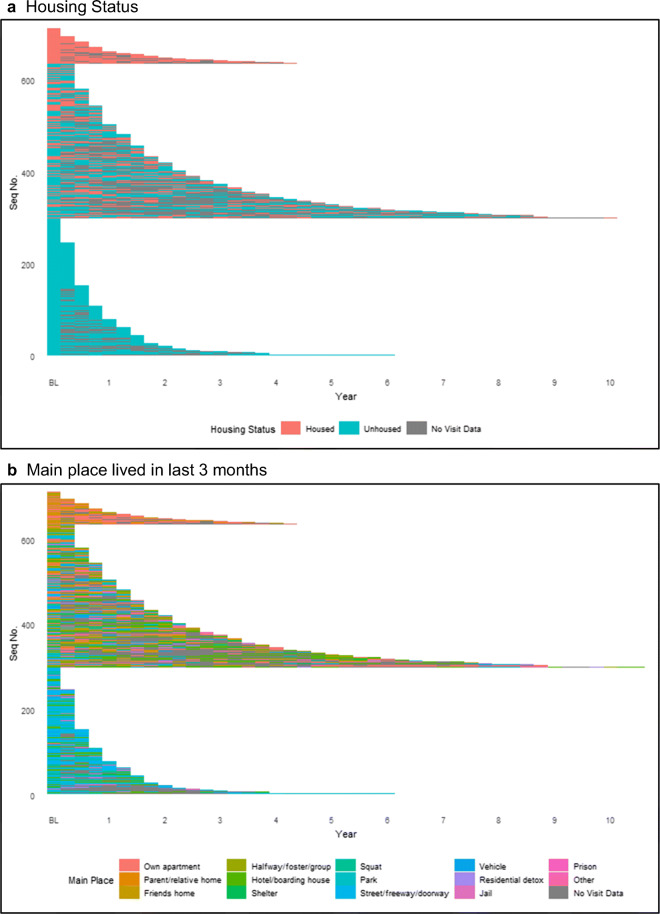
Longitudinal housing status in cohort participants, by quarter. **a** Housing status. **b** Main place lived in the past 3 months. Vertical axis represents individual participants, ordered by housing trajectory

The variability in housing status over time is described in Fig. 1. In Fig. 1a, only 77 participants (11%) reported being stably housed during their entire observation period. Two hundred ninety-eight participants (42%) were unhoused during their entire observation period. About half of participants (*n* = 337) moved between a “housed” and “unhoused” status, with a median number of 1 transition between housed and unhoused (IQR 1–3). Figure 1 b describes housing trajectories by the type of place, indicating a high frequency of transition between housing type over the observation period; the median number of transitions in housing type was 2 (IQR 1–4).

**Fig. 2:**
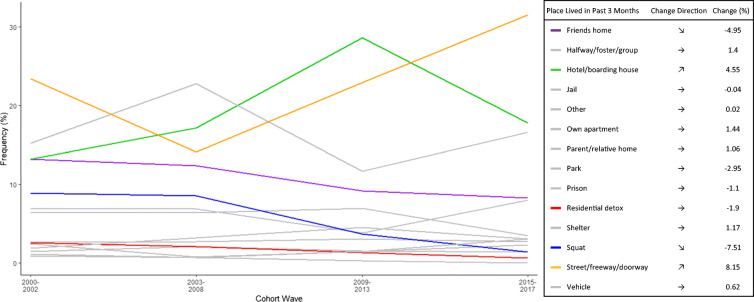
Frequency of main place lived in the past 3 months, by cohort wave

Figure 2 provides a population-level snapshot of the main housing type reported at baseline over calendar time (cohort waves). Little variability over time is seen in the proportion of participants reporting mainly living in their or parents’ home, half-way house, shelter, vehicle, residential detox, jail, or prison. A rapid decline in the proportion living in their friend’s apartment occurs in the 2003–2008 cohort wave, matched with a sharp increase in the proportion living on the street/freeway/doorway, which is retained during the later cohort waves. Similarly, in 2009–2013, a decline in the proportion living in temporary boarding hotels and increase in the proportion in residential detoxification services occur.

**Fig. 3:**
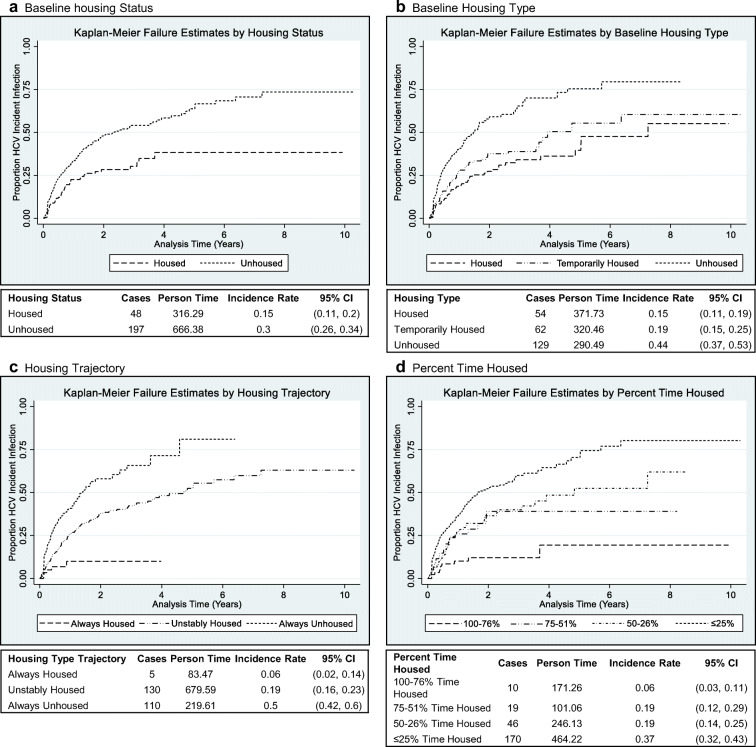
Four-panel figure of Kaplan-Meier curves with incidence rates listed below. **a** Baseline housing status. **b** Baseline housing type. **c** Housing trajectory. **d** Percent-time housed

Housing and incident HCV infection: Among the 712 participants, a total of 245 incident HCV infections were observed over 982.67 py, giving an overall HCV incidence estimate of 24.9 (95% confidence interval (CI) 21.9, 28.3) per 100 py. Across all measures of housing, the most unhoused group had the highest HCV incident infection rate (0.50, 95% CI 0.42, 0.60) (Fig. 3).

**Fig. 4:**
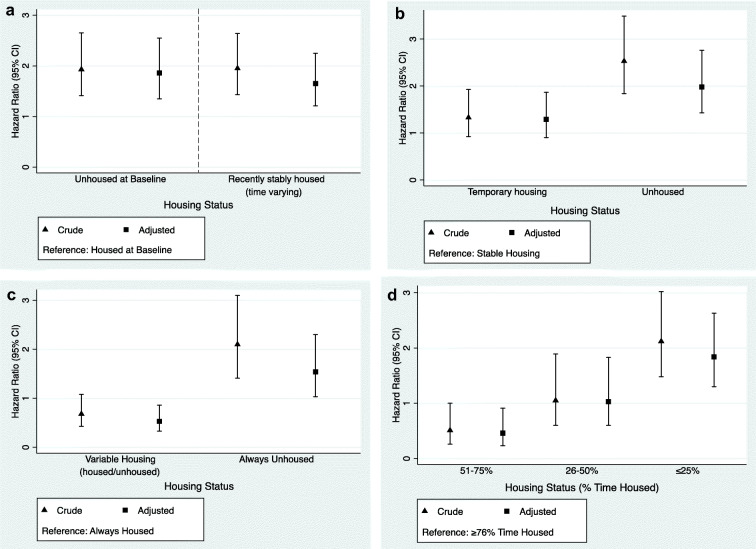
Unadjusted and adjusted Cox models with different exposure variables (*n* = 721). Variables included in the multivariable Cox models included age at enrollment, gender, recent injecting frequency*, recent needle/ancillary equipment sharing*, and number of recent injecting partners*. **a** Baseline housing status and recently housed (per 3 months). **b** Recent housing stability (per 3 months). **c** Housing status over time (cumulative). **d** Percent-time housed (cumulative). *Time-varying variable

In Cox proportional hazard regression models, we estimated aHR of HCV incident infection for several measures of being unhoused (relative to being housed), controlling for gender, age, injecting frequency, recent unsafe injecting behaviors, and number of injecting partners. When considering housing status, for the group unhoused at baseline, the aHR was 1.86 (95% CI 1.32, 2.55) and the aHR was 1.65 (95% CI 1.21, 2.25) for the time-varying measure. When considering housing stability, relative to those with stable housing, the aHR of HCV incident infection was 1.28 (95% CI 1.43, 2.76) among those unhoused and 1.29 (95% CI 0.90, 1.87) among those variably housed. When considering housing trajectory, relative to those housed throughout study observation, the aHR of HCV incident infection was 1.54 (95% CI 1.03, 2.30) for those unhoused throughout study observation and 0.53 (95% CI 0.33, 0.86) for those who moved between a housed and unhoused state. When considering percent-time housed, lower percent-time housed was associated with higher hazard. Compared with those housed for 75% or more of their study time, those housed less than 25% time had 1.84 times higher adjusted hazard (95% CI 1.30, 2.64), those housed 26–50% time had 1.03 times higher hazard (95% CI 0.60, 1.83), and those housed 51–75% time had 0.46 times the hazard (95% CI 0.23, 0.91) (Fig. 4).

## Discussion

Our findings from 15 years of study data support our hypothesis that young adult PWID experiencing both recent and chronic states of being unhoused are at elevated risk for HCV infection. Specifically, those who were unhoused at baseline had almost a twofold higher risk for HCV infection than their housed counterparts. And that with increased time being unhoused (a measure of chronic housing), HCV infection risk increased 80%, with those who were unhoused during all of their follow-up visits at one and a half greater risk for HCV infection. We observed a dose-response relationship between percent-time unhoused and risk for HCV infection, with a twofold increase in risk for HCV among young adult PWID housed less than 25% of the time compared with those housed at least 75% of the time. And that spending some percentage of time in stable housing provides protection from HCV infection. Given the current efforts to eliminate HCV as a public health burden [27, 28], our findings provide insight into the roles of recent and chronic housing instability for PWID which might impact the HCV epidemic. Our findings have practical implications for local and national HCV prevention initiatives and build upon the broader scientific understanding of housing and HCV risk in three main ways.

First, we encourage researchers to measure housing states at multiple time points to gain insights about individual’s larger housing trajectory. Although housing is measured in most observational research and conceptualized as a key social determinant of health, it is typically included in analysis as one-time, baseline measure and summarized in descriptive analysis of the study population without further investigation of its role in the main association of interest. Our inclusion of chronic housing–related risk measures recognizes the role of housing as a life course circumstance that can vary over time, with implications for health [29, 30]. We used repeated measures housing data to create multiple measures of housing to represent the period-specific and cumulative aspects of housing on HCV infection risk.

Results indicate that when looking beyond baseline binary housing status measures, young PWID experience varying patterns of housing over time. Over half moved between states of housed and unhoused living environments over time, including frequent periods of being unhoused: 89% of our sample experienced some duration of being unhoused with 42% unhoused the entirety of follow-up. Accounting for the temporal aspect of homeless experiences—such as whether someone is newly homeless versus chronically homeless—allows us to consider the ways where variation in housing instability could result in differential HCV risk. For example, among PWID, being unhoused may influence one’s ability to safely store injecting equipment leading to more frequent sharing of needles or ancillary equipment and higher risk for HCV infection [31–33]. Frequently relocating, as depicted in Figs. 1 and 2, can result in disruptions to employment, social networks, and receipt of social services including NSP services [34, 35]. Furthermore, by expanding measures to include housing trajectories, we were able to detect the protective benefits of being housed, even if only for a portion of the time.

Second, to our knowledge, this is the first study to assess the risk of HCV in association with housing in the USA. While numerous other researchers [31, 36–38] have demonstrated our finding that high rates of young adult PWID are unhoused, our detail of the heterogeneity in housing types (Fig. 1), the associations with HCV infection risk conferred by each housing type (Table 1), and how housing changes over time (Figs. 1 and 2) provide unique insight into lives of young adult PWID, the group with the highest incidence of HCV infection in the USA [39]. Our results indicate that even short intervals of being housed in a residence offer a protection to HCV infection. Additional information, via both quantitative and qualitative approaches, may help inform programmatic responses to expand protective qualities of being residentially housed. We intend, and hope others will too, to include new measures of privacy, safety, and security to deepen our understanding of the role housing type plays on health and wellbeing. Understanding housing among young PWID is important in the context of the HCV epidemic because youth experiencing homelessness have a harder time accessing services—including shelter, medical care, and employment—due to stigma, lack of knowledge of available resources, and limited availability of youth-focused services [40].

Third, by examining data over the 15-year period, we were able to demonstrate population-level changes in housing type over time. We observed an increase in the proportion of people living on the street, with a reduction in the proportion living in apartments. Housing is becoming more unstable across cities in the USA, leaving many young people vulnerable to acute and chronic homelessness [41]. At the same time, US cities are increasing municipal codes that disproportionately target street homeless [42]. In San Francisco, along with most US cities, bans on sitting, lying, and tent camping on the street and prohibitions on loitering and living in vehicles rose between 2006 and 2016 [43]. A qualitative study conducted in San Francisco demonstrated such punitive interactions, including move-along orders, systematically limit the ability of people living on the street to access services and increase intra-neighborhood mobility [44]. This combined with the criminalization of drug use heightens police targeting of unhoused PWID [45], and increases occurrence of risky injecting practices associated with HCV and HIV [46, 47].

Our study results should be considered in the context of various limitations. One limitation relates to the study setting of San Francisco. The rapidly evolving demographics and gentrification San Francisco has experienced may complicate interpretations of our findings. In addition, San Francisco is a particularly high-resource setting for housing support and harm reduction programs for PWID, which may bias our results toward the null. Further, we relied on self-reported data for exposure and covariates subject to recall and social desirability bias. Researchers should use caution when comparing results with other studies. Despite sensitivity analyses suggesting that results remained relatively unchanged, with the direction and magnitude of associations retained, when not restructuring data to a fit the 3-month follow-up schedule, it is still possible that using the last value carryforward method bias results away from the null. Next, results from our sensitivity analysis comparing the analytic sample with those lost to follow-up noted that those retained were significantly older and displayed less risky injecting behaviors than those lost to follow-up and therefore likely biased our results toward the null resulting in smaller effect sizes. Finally, the association between being unhoused and incident HCV infection is likely complex. Opportunity exists to expand the measurement dimensions for housing to capture additional elements the housing space. Future studies of housing and HCV incidence could incorporate innovative methodological strategies, such as GPS tracking [48], to understand the frequency and patterns of spatial movements and contextual attributes. Such studies would enable a more comprehensive understanding of recent and chronic housing by considering the more nuanced ways that housing characteristics impact behaviors and wellbeing.

## Electronic Supplementary Material

ESM 1 (DOCX 44 kb)

## Abbreviations

aHR: adjusted hazard ratio
CI: confidence interval
HCV: hepatitis C virus
HR: hazard ratio
PWID: people who inject drugs
PY: person-years

## References

[1] Busch-Geertsema V, Culhane D, Fitzpatrick S (2016). Developing a global framework for conceptualising and measuring homelessness. Habitat Int.

[2] Shaw M. Housing and public health. 2004. 10.1146/annurevpublhealth25101802123036.

[3] Aidala AA, Wilson MG, Shubert V, et al. Housing status, medical care, and health outcomes among people living with HIV/AIDS: a systematic review. 2015. 10.2105/AJPH2015302905. 10.2105/AJPH.2015.302905 PMC4695926 26562123

[4] Stahre M, Van Eenwyk J, Siegel P, Njai R. Housing insecurity and the association with health outcomes and unhealthy behaviors, Washington State, 2011. *Prev Chronic Dis.* 2015;12. 10.5888/pcd12.140511 PMC4509099 26160295

[5] Krieger J, Higgins DL (2002). Housing and health: time again for public health action. Am J Public Health.

[6] Schanzer B, Dominguez B, Shrout PE, Caton CLM. Homelessness, health status, and health care use. 2011. 10.2105/AJPH2005076190. 10.2105/AJPH.2005.076190 PMC1805022 17267724

[7] Chau S, Chin M, Chang J (2002). Cancer risk behaviors and screening rates among homeless adults in Los Angeles County. Cancer Epidemiol Biomark Prev.

[8] *Homelessness during pregnancy: a unique, time-dependent risk factor of birth outcomes.* SpringerLink; 2019. 10.1007/s10995-014-1633-6 25404405

[9] *Housing instability and birth weight among young urban mothers.* SpringerLink; 2019. 10.1007/s11524-014-9913-4 PMC4338127 25344356

[10] Simon AE, Fenelon A, Helms V, Lloyd PC, Rossen LM (2017). HUD housing assistance associated with lower uninsurance rates and unmet medical need. Health Aff.

[11] Brown RT, Miao Y, Mitchell SL, Bharel M, Patel M, Ard KL, Grande LJ, Blazey-Martin D, Floru D, Steinman MA (2015). Health outcomes of obtaining housing among older homeless adults. Am J Public Health.

[12] Larimer ME, Malone DK, Garner MD, Atkins DC, Burlingham B, Lonczak HS, Tanzer K, Ginzler J, Clifasefi SL, Hobson WG, Marlatt GA (2009). Health care and public service use and costs before and after provision of housing for chronically homeless persons with severe alcohol problems. JAMA.

[13] Collins SE, Malone DK, Clifasefi SL, Ginzler JA, Garner MD, Burlingham B, Lonczak HS, Dana EA, Kirouac M, Tanzer K, Hobson WG, Marlatt GA, Larimer ME (2012). Project-based Housing First for chronically homeless individuals with alcohol problems: within-subjects analyses of 2-year alcohol trajectories. Am J Public Health.

[14] Hofmeister MG, Rosenthal EM, Barker LK, Rosenberg ES, Barranco MA, Hall EW, Edlin BR, Mermin J, Ward JW, Ryerson AB (2019). Estimating prevalence of hepatitis C virus infection in the United States, 2013-2016. Hepatology.

[15] Facente SN, Grebe E, Burk K, Morris MD, Murphy EL, Mirzazadeh A, Smith AA, Sanchez MA, Evans JL, Nishimura A, Raymond HF, on behalf of End Hep C SF (2018). Estimated hepatitis C prevalence and key population sizes in San Francisco: a foundation for elimination. PLoS One.

[16] Centers for Disease Control and Prevention, (CDC). *Who are homeless, individuals: current statistics on the prevalence and characteristics of people experiencing homelessness in the United States.*

[17] Parmar P, Corsi DJ, Cooper C (2016). Distribution of hepatitis C risk factors and HCV treatment outcomes among Central Canadian aboriginal. Can J Gastroenterol Hepatol.

[18] Sacks-Davis R, Aitken CK, Higgs P, et al. High rates of hepatitis C virus reinfection and spontaneous clearance of reinfection in people who inject drugs: a prospective cohort study. *PLoS One.* 2013;8. 10.1371/journal.pone.0080216 PMC3820644 24244654

[19] Rourke SB, Sobota M, Tucker R, et al. Social determinants of health associated with hepatitis C co-infection among people living with HIV: results from the Positive Spaces, Healthy Places study. *Open Med.* 2011;5:e120–131. PMC3205830 22046224

[20] Kim C, Kerr T, Li K, Zhang R, Tyndall MW, Montaner JSG, Wood E (2009). Unstable housing and hepatitis C incidence among injection drug users in a Canadian setting. BMC Public Health.

[21] Patterson ML, Somers JM, Moniruzzaman A (2012). Prolonged and persistent homelessness: multivariable analyses in a cohort experiencing current homelessness and mental illness in Vancouver, British Columbia. Ment Health Subst Use.

[22] Burt MR (2002). Chronic homelessness: emergence of a public policy. Fordham Urb LJ.

[23] Housing Instability Research Department (HIRD). *SAN FRANCISCO HOMELESS COUNT & SURVEY 2017, comprehensive report.* http://hsh.sfgov.org/wp-content/uploads/2017/06/2017-SF-Point-in-Time-Count-General-FINAL-6.21.17.pdf2017.

[24] Page K, Hahn JA, Evans J, Shiboski S, Lum P, Delwart E, Tobler L, Andrews W, Avanesyan L, Cooper S, Busch MP (2009). Acute hepatitis C virus infection in young adult injection drug users: a prospective study of incident infection, resolution, and reinfection. J Infect Dis.

[25] Hahn JA, Page-Shafer K, Lum PJ, Bourgois P, Stein E, Evans JL, Busch MP, Tobler LH, Phelps B, Moss AR (2002). Hepatitis C virus seroconversion among young injection drug users: relationships and risks. J Infect Dis.

[26] Page-Shafer K, Pappalardo BL, Tobler LH, Phelps BH, Edlin BR, Moss AR, Wright TL, Wright DJ, O’Brien TR, Caglioti S, Busch MP (2008). Testing strategy to identify cases of acute hepatitis C virus (HCV) infection and to project HCV incidence rates. J Clin Microbiol.

[27] World Health Organization (WHO). *Combating hepatitis B and C to reach elimination by 2030: advocacy brief*. 2016.

[28] Grebely J, Bruneau J, Lazarus JV, Dalgard O, Bruggmann P, Treloar C, Hickman M, Hellard M, Roberts T, Crooks L, Midgard H, Larney S, Degenhardt L, Alho H, Byrne J, Dillon JF, Feld JJ, Foster G, Goldberg D, Lloyd AR, Reimer J, Robaeys G, Torrens M, Wright N, Maremmani I, Norton BL, Litwin AH, Dore GJ, International Network on Hepatitis in Substance Users (2017). Research priorities to achieve universal access to hepatitis C prevention, management and direct-acting antiviral treatment among people who inject drugs. Int J Drug Policy.

[29] Desmond M. *Evicted: poverty and profit in the American City*: Broadway Books; 2016.

[30] Díaz MCE (2017). Rented, crowded, and unaffordable? Social vulnerabilities and the accumulation of precarious housing conditions in Los Angeles. Hous Policy Debate.

[31] Bozinoff N, Wood E, Dong H, Richardson L, Kerr T, DeBeck K (2017). Syringe sharing among a prospective cohort of street-involved youth: implications for needle distribution programs. AIDS Behav.

[32] Des Jarlais DC, Braine N, Friedmann P (2007). Unstable housing as a factor for increased injection risk behavior at US syringe exchange programs. AIDS Behav.

[33] Pilarinos A, Kennedy MC, McNeil R, Dong H, Kerr T, DeBeck K (2017). The association between residential eviction and syringe sharing among a prospective cohort of street-involved youth. Harm Reduct J.

[34] Martinez AN, Lorvick J, Kral AH (2014). Activity spaces among injection drug users in San Francisco. Int J Drug Policy.

[35] Szreter S, Woolcock M (2004). Health by association? Social capital, social theory, and the political economy of public health. Int J Epidemiol.

[36] Craine N, Hickman M, Parry JV (2009). Incidence of hepatitis C in drug injectors: the role of homelessness, opiate substitution treatment, equipment sharing, and community size. Epidemiol Infect.

[37] Rhodes T, Treloar C (2008). The social production of hepatitis C risk among injecting drug users: a qualitative synthesis. Addiction..

[38] Smereck GA, Hockman EM (1998). Prevalence of HIV infection and HIV risk behaviors associated with living place: on-the-street homeless drug users as a special target population for public health intervention. Am J Drug Alcohol Abuse.

[39] Suryaprasad AG, White JZ, Xu F, Eichler BA, Hamilton J, Patel A, Hamdounia SB, Church DR, Barton K, Fisher C, Macomber K, Stanley M, Guilfoyle SM, Sweet K, Liu S, Iqbal K, Tohme R, Sharapov U, Kupronis BA, Ward JW, Holmberg SD (2014). Emerging epidemic of hepatitis C virus infections among young nonurban persons who inject drugs in the United States, 2006-2012. Clin Infect Dis.

[40] National Coalition for the Homeless. Homeless Youth Fact Sheet. 2008. http://www.nationalhomeless.org/factsheets/youth.html. Accessed 26 Feb 2019.

[41] Morton MH, Dworsky A, Matjasko JL, Curry SR, Schlueter D, Chávez R, Farrell AF (2018). Prevalence and correlates of youth homelessness in the United States. J Adolesc Health.

[42] Bauman T, Rosen J, Tars E, Foscarinis M, Fernandea J. No safe place: the criminalization of homelessness in US cities. *National Law Center on Homelessness & Poverty*; 2014.

[43] Robinson T (2019). No right to rest: police enforcement patterns and quality of life consequences of the criminalization of homelessness. Urban Aff Rev.

[44] Herring C, Yarbrough D, Marie Alatorre L. Pervasive penality: how the criminalization of poverty perpetuates homelessness. *Social Problems.* 2019.

[45] Kerr T, Small W, Wood E (2005). The public health and social impacts of drug market enforcement: a review of the evidence. Int J Drug Policy.

[46] Wagner KD, Pollini RA, Patterson TL, Lozada R, Ojeda VD, Brouwer KC, Vera A, Volkmann TA, Strathdee SA (2011). Cross-border drug injection relationships among injection drug users in Tijuana, Mexico. Drug Alcohol Depend.

[47] Werb D, Wood E, Small W, Strathdee S, Li K, Montaner J, Kerr T (2008). Effects of police confiscation of illicit drugs and syringes among injection drug users in Vancouver. Int J Drug Policy.

[48] Mirzazadeh A, Grasso M, Johnson K, Briceno A, Navadeh S, McFarland W, Page K (2014). Acceptability of Global Positioning System technology to survey injecting drug users’ movements and social interactions: a pilot study from San Francisco, USA. Technol Health Care.

