# Supplementary material for: Housing Stability and Hepatitis C Infection for Young Adults Who Inject Drugs: Examining the Relationship of Consistent and Intermittent Housing Status on HCV Infection Risk
Source: J Urban Health. 2020 Sep 8;97(6):831–44. doi: 10.1007/s11524-020-00445-7 (PMC7704865; doi:10.1007/s11524-020-00445-7)
Supplement: Supplementary file 1 — (DOCX 44 kb) [file 11524_2020_445_MOESM1_ESM.docx]

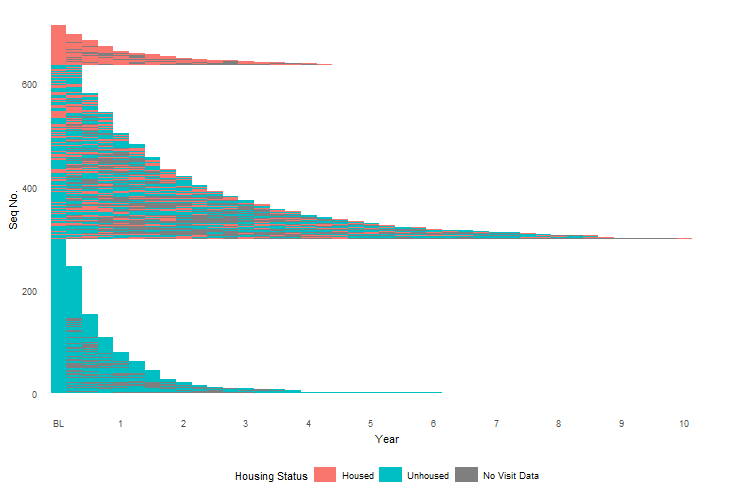

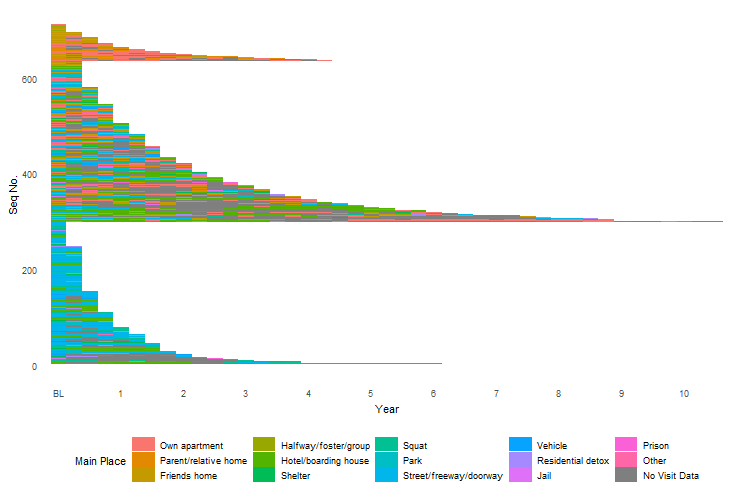

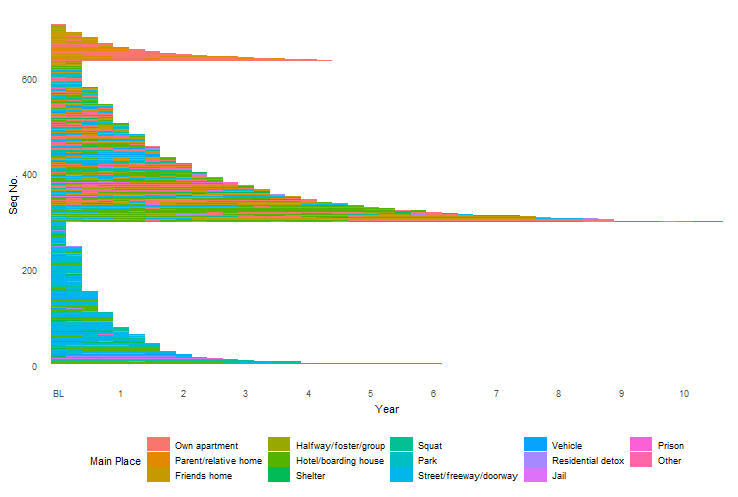

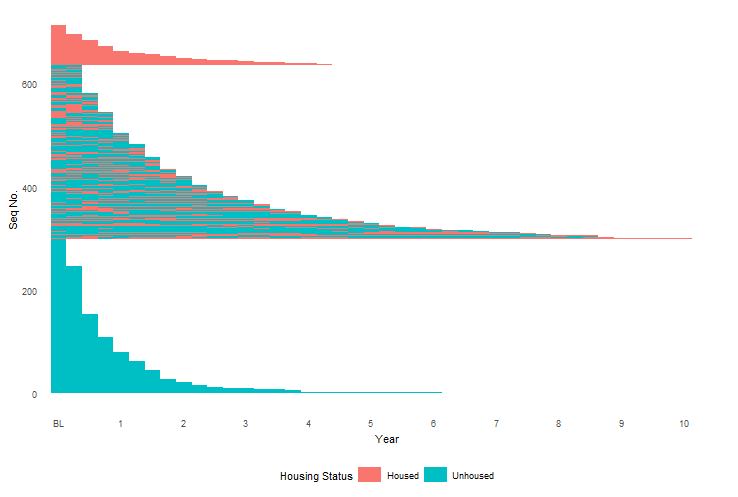


Panel S1c: Main place lived in last 3 months

Panel S1a: Housing Status

Supplemental Figure 1: Longitudinal housing status in UFO cohort participants by quarter.

Panel 3b: Housing Status, Imputed

Panel 3d: Main place lived in last 3 months, Imputed
